# The Critical Role of Growth Factors in Gastric Ulcer Healing: The Cellular and Molecular Mechanisms and Potential Clinical Implications

**DOI:** 10.3390/cells10081964

**Published:** 2021-08-02

**Authors:** Andrzej S. Tarnawski, Amrita Ahluwalia

**Affiliations:** 1 Medical Research Service, VA Long Beach Healthcare System Long Beach, 5901 East Seventh Street, Long Beach, CA 90822, USA; 2 Division of Gastroenterology, Department of Medicine and Digestive Health Institute, The University of California-Irvine, Irvine, CA 92697, USA

**Keywords:** gastric ulcer healing, growth factors, signaling pathways, angiogenesis, vasculogenesis, bone marrow derived endothelial progenitor cells

## Abstract

In this article we review the cellular and molecular mechanisms of gastric ulcer healing. A gastric ulcer (GU) is a deep defect in the gastric wall penetrating through the entire mucosa and the muscularis mucosae. GU healing is a regeneration process that encompasses cell dedifferentiation, proliferation, migration, re-epithelialization, formation of granulation tissue, angiogenesis, vasculogenesis, interactions between various cells and the matrix, and tissue remodeling, all resulting in scar formation. All these events are controlled by cytokines and growth factors (e.g., EGF, TGFα, IGF-1, HGF, bFGF, TGFβ, NGF, VEGF, angiopoietins) and transcription factors activated by tissue injury. These growth factors bind to their receptors and trigger cell proliferation, migration, and survival pathways through Ras, MAPK, PI3K/Akt, PLC-γ, and Rho/Rac/actin signaling. The triggers for the activation of these growth factors are tissue injury and hypoxia. EGF, its receptor, IGF-1, HGF, and COX-2 are important for epithelial cell proliferation, migration, re-epithelialization, and gastric gland reconstruction. VEGF, angiopoietins, bFGF, and NGF are crucial for blood vessel regeneration in GU scars. The serum response factor (SRF) is essential for VEGF-induced angiogenesis, re-epithelialization, and blood vessel and muscle restoration. Local therapy with cDNA of human recombinant VEGF165 in combination with angiopoietin1, or with the NGF protein, dramatically accelerates GU healing and improves the quality of mucosal restoration within ulcer scars. The future directions for accelerating and improving healing include local gene and protein therapies with growth factors, their combinations, and the use of stem cells and tissue engineering.

## 1. Overview of Gastric Ulcer

**Figure 1:**
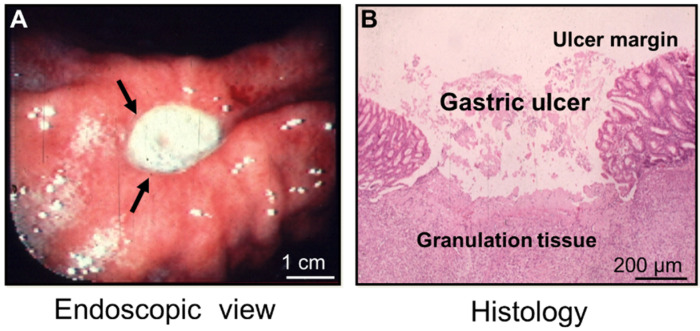
A gastric ulcer—endoscopic view and histology (reprinted with permission from [1]). (**A**) An endoscopic picture of a human gastric ulcer on the lesser curvature of the stomach (arrows). The ulcer is covered with white fibrinopurulent material. (**B**) A histological image of an experimental gastric ulcer in a rat model; hematoxylin and eosin (H&E) staining were used.

In this article we review the cellular and molecular mechanisms of gastric ulcer (GU) healing with a focus on the critical role of growth factors in this process. A gastric ulcer is a deep defect in the gastric wall penetrating through the entire mucosa and the muscularis mucosae (Figure 1—modified and reprinted with permission from [1]).

**Figure 2:**
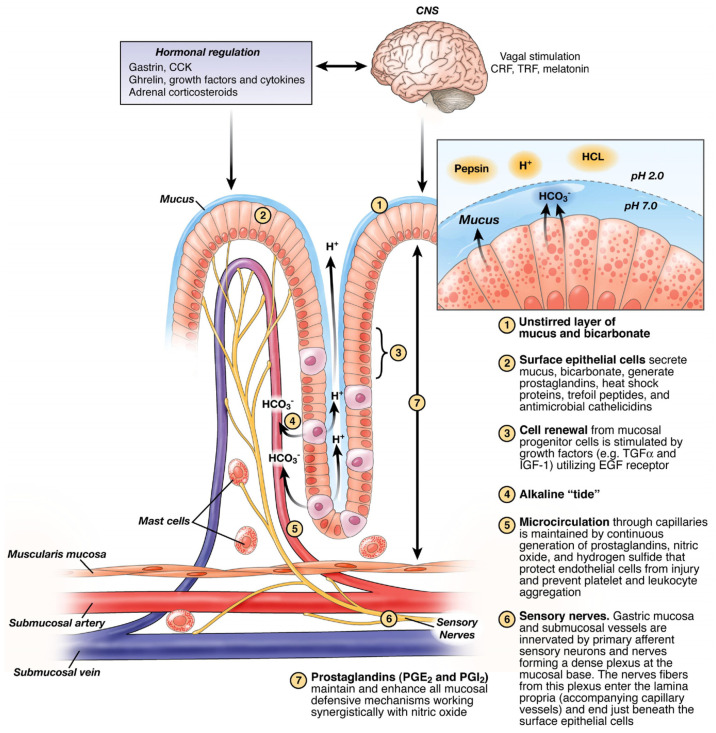
Mucosal defense. A diagrammatic representation of gastric mucosal defense (designed by A. Tarnawski; reprinted with permission from [4]).

The ulcer develops when the mucosal defense (Figure 2) is overwhelmed by noxious factors such as *H. pylori* infection, NSAIDs and/or gastric acid hypersecretion. The tissue necrosis is caused by vascular and microvascular injury, resulting in mucosal ischemia, hypoxia, cessation of nutrient and oxygen delivery, and free radical formation [2]. Tissue necrosis and the release of leukotriene B attract leukocytes and macrophages, which phagocytize necrotic tissue and release pro-inflammatory cytokines, e.g., TNFα, IL-lα, and IL-lβ. They in turn activate local fibroblasts, endothelial cells, and epithelial cells [3].

**Figure 3:**
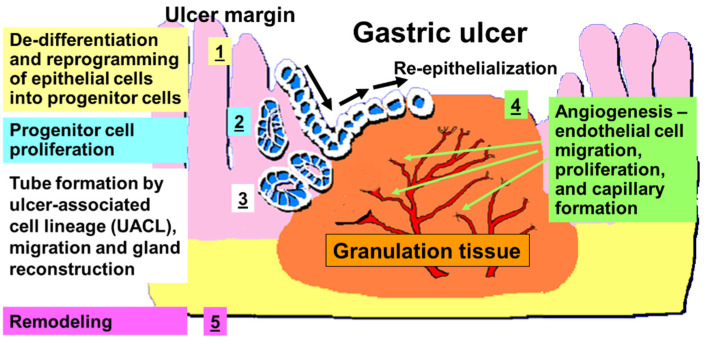
A diagrammatic representation of GU healing: re-epithelization, gland reconstruction, and angiogenesis. Modified from A. S. Tarnawski’s state-of-the-art presentation at EURO Symposium at the Pasteur Institute, Paris in 2005.

Histologically, two major structures can be identified in GU: the ulcer margin, non-necrotic mucosa bordering necrosis—the epithelial component; and the granulation tissue at the ulcer’s base—the connective tissue component (Figure 1) [5,6,7]. The latter consists of fibroblasts, macrophages, and proliferating endothelial cells forming blood vessels and microvessels. The healing of a GU is a regeneration process that includes cell proliferation, migration, re-epithelialization, formation of granulation tissue, angiogenesis, and interactions among various cells and the matrix, all resulting in scar formation and tissue remodeling (Figure 3) [2,3,5,6,7,8,9,10,11]. All these processes are controlled by growth factors, transcription factors, and cytokines [5,7,10,12,13].

## 2. Cellular and Molecular Events in the Ulcer Margin

The mucosa of the ulcer margin forms a distinct “healing zone” (Figure 3) [5,6,7]. The gastric glands in this zone become dilated; the parietal and chief cells are mostly absent, because of their reprogramming toward progenitor cells [14]. These reprogrammed cells express epidermal growth factor receptor (EGF-R, a marker of gastric progenitor cells) and actively proliferate [5,7,15]. The latter process is initiated within 2–3 days after ulcer formation and is essential for ulcer healing, because it supplies epithelial cells crucial for migration and the re-epithelialization of mucosal surface, and the reconstruction of gastric glands [5,7,11]. These reprogrammed cells migrate from the ulcer margin onto the granulation tissue to re-epithelialize the ulcer base, as shown in Figure 3. In addition, the epithelial cells from the base of the ulcer margin form tubes of “ulcer-associated cell lineage” (UACL), which invade granulation tissue, migrate toward the surface, branch, and undergo transformation into gastric glands within the ulcer scar (Figure 3) [5,11,16].

The major stimuli for cell proliferation, division, migration, and re-epithelialization are growth factors [5,6,10,12,13]. In addition to the initial pool of growth factors derived from the platelets, macrophages, and injured tissue, gastric ulceration triggers in cells lining the mucosa of the ulcer margin, activation of genes encoding for growth factors (e.g., EGF, TGFα, bFGF, HGF, and trefoil peptides) and COX-2 in a well synchronized spatial and temporal manner [7,13,17]. These growth factors, which are produced locally, activate epithelial cell migration and proliferation via autocrine and/or paracrine actions. Some studies indicate that in addition to utilizing local cells from the mucosa of the ulcer margin, the epithelium of damaged or ulcerated mucosa can be regenerated by bone marrow-derived adult stem cells [18]. Okamoto and co-workers demonstrated that bone marrow cells can re-populate epithelium of human gastrointestinal tract, and this process is increased by many fold in GU [18]. Re-epithelialization, the migration of epithelial cells from the ulcer margin to restore epithelial continuity, is an essential process for cutaneous and gastrointestinal wound/ulcer healing (Figure 3) [5,6,10,11,12]. This process is essential because a continuous epithelial barrier is critical for protecting granulation tissue and newly formed blood vessels from injury by luminal noxious factors.

The 2012 Nobel Prize in Medicine/Physiology was awarded to Sir John B. Gurdon and Shinya Yamanaka for their discovery that mature, specialized cells can de-differentiate into progenitor/stem cells [19,20]. This novel concept was predominantly developed in in vitro models and has not been examined during GU healing. Our recent study indicates that de-differentiation and reprogramming of epithelial cells toward progenitor cells occur in adult tissue in response to injury in GU margin and are triggered by hypoxia, and activation of EGF-R expression and signaling [14]. The main argument supporting this contention is the fact that mature, specialized parietal and chief cells in the mucosa of GU margin “disappear” without any signs of apoptosis, and thus are changed to cells strongly expressing EGF-R—the marker of progenitor cells in human and rat gastric mucosa. This concept requires further research and confirmation, but it is supported by other studies showing that EGF-R activity is required for renal tubular cell dedifferentiation and proliferation in a murine model of folic acid-induced acute kidney injury [21,22].

## 3. Growth Factors—Discovery, Brief Historical Background, and Their Role in GU Healing

The first two growth factors discovered in the 1950s were epidermal growth factor (EGF) and nerve growth factor (NGF) [23,24]. EGF was discovered by Professor Stanly Cohen, who worked with Professor Rita Levi-Montalcini at the Washington University in St. Louis during search to identify NGF. For these discoveries, Levi-Montalcini and Cohen were awarded the 1986 Nobel Prize in Physiology/Medicine [23,24,25,26]. Both these growth factors were discovered in salivary glands in mice and subsequently identified in other cells and tissues [25,27,28]. EGF binds to its receptor, EGF-R, and stimulates cell migration, proliferation, and differentiation [17]. Professor Cohen attended our International Workshop on The Cellular and Molecular Mechanisms of Ulcer Healing in Antalya Turkey in 1993 and presented the history of EGF discovery [25].

EGF acts by binding with high affinity to epidermal growth factor receptor (EGF-R) on the cell surface. This event stimulates ligand-induced dimerization [23,24,25], activating the intrinsic protein-tyrosine kinase activity of the receptor. The tyrosine kinase activity, in turn, initiates a signal transduction cascade that results in a variety of biochemical changes within the cell—a rise in intracellular calcium levels; increased glycolysis and protein synthesis; and increases in the expression of certain genes, including the gene for EGF-R, that ultimately lead to DNA synthesis and cell proliferation [29,30]. EGF is the fundamental member of the EGF-family of proteins. Members of this protein family have highly similar structural and functional characteristics. Besides, EGF itself other family members include heparin-binding EGF-like growth factor (HB-EGF), transforming growth factor-α (TGFα), amphiregulin (AR), epiregulin (EPR), epigen, betacellulin (BTC), and neuregulins 1-4 (NRG 1-4). In normal gastric mucosa, EGF-R is expressed in few progenitor cells in the neck area of gastric gland, and the normal proliferation is driven predominantly by TGFα, which is also an EGF-R ligand. In response to a mucosal injury such as a GU, luminal EGF originating from salivary glands and secreted locally by progenitor cells contributes to EGF-R activation. HIF-1α was shown to activate EGF-R in chronic obstructive pulmonary disease [31]. Regarding gastrointestinal epithelial cells, Basson et al. demonstrated in an in vitro study that EGF stimulates enterocyte migration by modulating extracellular matrix and integrin expression and organization [27,32]. Importantly, this study showed that in vitro injury can induce the modulation of these cells or trans-differentiation [27,32].

In the study of human ulcer specimens, Wright and coworkers showed that ulceration in the human gastrointestinal tract induces the development of a novel cell lineage, the “ulcer-associated cell lineage” (UACL), which likely originates from gastrointestinal stem cells [16]. This cell lineage secreting EGF buds from the ulcer margin, grows locally forming new glands, and serves to regenerate mucosal structures during ulcer healing. This cell lineage is commonly associated with gastrointestinal mucosal ulceration, and Wright and coworkers concluded that the principal in vivo role of EGF is to stimulate ulcer healing in the gut through induction of this cell lineage in the adjacent mucosa.

**Figure 4:**
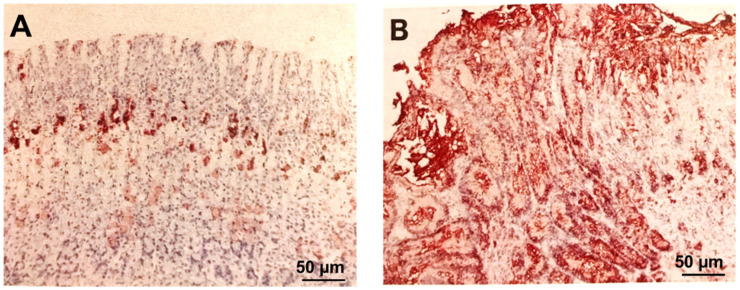
Photomicrographs of rat gastric oxyntic mucosa immunostained for epidermal growth factor receptor and counterstained with hematoxylin. (**A**) Normal rat gastric oxyntic mucosa shows epidermal growth factor receptor localization only in some neck cells of gastric glands (the progenitor zone; brown staining). (**B**) Mucosa at the GU margin 7 days after ulcer induction shows the “disappearance” of parietal and chief cells due to their dedifferentiation and reprogramming to progenitor cells (brown staining) through increased expression of epidermal growth factor receptors in the majority of cells lining GU margin. The cells demonstrating epidermal growth factor receptor expression occupy the entire mucosal thickness (reprinted with permission from [15]).

Our laboratory demonstrated that gastric ulceration in rats dramatically increases expression of EGF-R in the mucosa of the GU margin. In mucosa of the GU margin, at 7 and 16 days after ulcer induction, there was a 75-fold increase (over controls) in the number of cells expressing EGF-R [14]. The percentage of total area of mucosal section occupied by cells positively stained for EGF-R was 0.51 ± 0.1% in normal control mucosa, and the results were 38.8 ± 3%, 37.8 ± 2%, 37.2 ± 2%, and 38.3 ± 2%, respectively, in the mucosa of the ulcer margin or ulcer scar after 3, 7, 16, and 25 days, respectively (all *p* < 0.001 vs. mucosa of normal controls or sham-operated rat (Figure 4) [14].

## 4. The Role of EGF in GU Healing

Brzozowski et al. showed that local treatment with EGF produced a significant decrease in gastric acid secretion, increased the expression of COX-2, and significantly accelerated the rate of ulcer healing in a rat model [33]. The acceleration of ulcer healing and hyperemia at the ulcer margin exhibited by locally applied EGF were similar to the results obtained after systemic administration of this growth factor [33]. Anti-EGF antibody completely abolished the acceleration of the ulcer healing at the ulcer margin induced by this growth factor [33]. These studies clearly demonstrated the GU healing effect of EGF. However, the cellular and molecular targets, mechanisms, and signaling pathways remained unelucidated. More recent studies identified some of these cellular and molecular targets for gastric ulcer healing. The cellular events of GU healing at the ulcer margin include reprogramming of majority of cells to the progenitor lineage, cell proliferation, migration, the development of UACL, and gland reconstruction. The molecular events at the ulcer margin include the activation of genes encoding EGF-R and EGF, IGF-1, Trefoil peptides, PDGF, HGF and c-met/HGF-R, COX-2, c-fos, c-jun, egr-1, SP-1, and other growth factors and cytokines.

**Figure 5:**
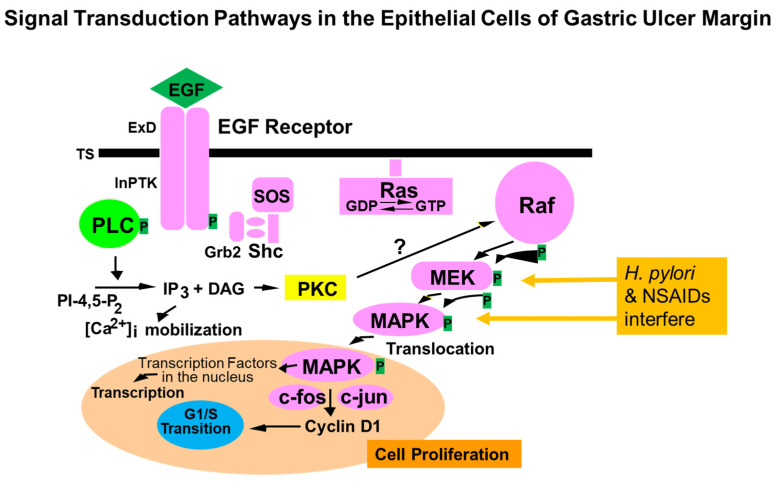
Signaling pathway induced by the activation of EGF-R in gastric epithelial cells in the mucosa of the ulcer margin. NSAIDs and H. pylori interfere with this signaling pathway and inhibit ulcer healing.

Activation of COX-2 by EGF has significant implications because COX-2 generates prostaglandins, e.g., PGE2 can protect regenerating cells by cytoprotective mechanisms [4]. In addition, PGE2 was shown to transactivate EGF-R by activating a matrix metalloproteinase that in turn releases TGFα, an alternative ligand for EGF-R [34]. Our previous study demonstrated that gastric ulceration, in epithelial cells of the ulcer margin, activates the EGF-R/ERK signaling cascade, which is reflected by increased tyrosine kinase activity, increased EGF-R protein and phosphorylation, and ERK1 and ERK2 phosphorylation and activity [35]. Additionally, epithelial cells isolated from GU margins showed significant increases in ERK1 and ERK2 activity. Furthermore, that study showed that treatment with tyrphostin A46 (inhibitor of EGF-R kinase and EGF-R kinase-dependent cell proliferation) significantly delays ulcer healing and reduces EGF-R expression, its phosphorylation, and ERK1 and ERK2 activity. That study, in addition to showing increased receptor tyrosine kinase activity, EGF-R levels, and EGF-R phosphorylation, delineated important mediators of signal transduction involved in gastric ulcer healing, namely, extracellular signal-regulated kinases ERK1 and ERK2. These data also showed that during healing of GU, the downstream events after activation of EGF-R tyrosine kinase enzyme activity and phosphorylation of ERK1 and ERK2 [35]. In a follow-up study aimed at the cellular and molecular mechanisms of EGF-promoted GU healing action, we demonstrated that during experimental GU healing, Raf-1 activity is increased, and that Raf-1 activation involves increased Shc-Grb2-Sos association. Furthermore, that study suggested that ERK activation during ulcer healing is not mediated by PKC. Similarly, treatment of rat gastric epithelial cells with EGF increased Ras and ERK activity, but not PKC’s activity. Thus, activation of the Raf-1–ERK cascade during gastric ulcer healing is Ras-mediated (Figure 5) and is attributable to the epithelial component of ulcer margins [36]. *H. pylori* and NSAIDs interfere with MEK and MAPK phosphorylation and block these pathways (Figure 5).

Transforming growth factor alpha (TGFα) is another EGF-R ligand. Yetkin et al. showed that microemulsion and an aqueous solution containing TGFα and/or aprotinin administered intragastrically accelerate healing of acute aspirin induced GUs [37].

## 5. Trefoil Factor (TFF) Peptides and GU Healing

TFF peptides are a family of mucin-associated secretory molecules that contain a 40-amino acid motif, including six conserved cysteine residues [38,39,40]. There are three mammalian trefoil factor (TFF) peptides: TFF1 (original name: pS2), TFF2 (original name; pancreatic spasmolytic polypeptide, PSP), and TFF3 (original name; intestinal trefoil factor, ITF) [39,40]. TFF peptides play important roles in response to GI mucosal injury and inflammation [41]. In response to acute GI mucosal injury, TFF peptides accelerate cell migration to seal the damaged area from luminal contents [38]. They protect gastric mucosa against injury by NSAIDs or ethanol in rats and human cell lines and regulate cell survival via ERK/MAPK, PI3K/Akt, phospholipase C (PLC)/PKC, β-catenin, and EGF signaling pathway [38]. TFF3 upregulation was found in mouse ulcer models and human ulcer patients, and was often associated with intestinal metaplasia [38]. TFF1 and TFF2 proteins are both upregulated and appear in the regenerating glands of healing GUs [38]. The appearance of the TFF2-expressing lineage is coincident with the loss of parietal cells and is referred to as spasmolytic polypeptide expressing metaplasia (SPEM). Gastric ulcer healing is dramatically delayed in TFF2 KO mice [42], and slow cell migration may be exacerbated by losing the effects of TFF peptides on proliferation or cell survival [12,43,44].

## 6. HGF and GU Healing

Hepatocyte growth factor (HGF) or scatter factor (SF), discovered in the mid-1980s as a hepatotrophic factor, is a cellular growth, motility, and morphogenic factor for numerous cells [45]. It is secreted by mesenchymal cells and acts mainly on epithelial and endothelial cells, haemopoietic progenitor cells, and T cells. Brzozowski et al. showed that local treatment with HGF significantly accelerates GU healing, increases blood flow at the ulcer margin, and upregulates COX-2 expression in the ulcerated mucosa [33]. The acceleration of ulcer healing and hyperemia at the ulcer margin exhibited by locally applied HGF occurred to a similar extent as with systemic administration of these growth factors. Additionally, HGF when applied submucosally upregulated COX-2 expression, and this action was significantly reversed by concurrent treatment with antibody against this peptide [33]. A separate study showed that HGF triggers activation of the COX-2 gene in gastric epithelial cells through phosphorylation of c-Met/HGF receptor and activation of the ERK2 signaling pathway [46].

## 7. Fibroblast Growth Factors and GU Healing

Acidic FGF (FGF1) and basic FGF (bFGF; FGF2) were discovered in 1973 [47,48]. They were originally isolated from the brain and pituitary gland as growth factors for fibroblasts, but they are expressed in a variety of cells and tissues. Since then, at least 22 distinct FGFs have been identified or isolated. Basic fibroblast growth factor (bFGF or FGF-2) promotes growth of mesenchymal and epithelial cells and stimulates angiogenesis and neuroprotection. Exogenous bFGF promotes healing of gastroduodenal ulcers [49]. All these actions were demonstrated regarding the 18 kDa bFGF isoform that is secreted by cells via an ER/Golgi-independent pathway and activates FGF receptors. Ernst and coworkers demonstrated that local injection of bFGF into the GU site accelerates GU healing, angiogenesis, and local gastric blood flow, whereas a neutralizing antibody of bFGF had the opposite effects [50]. Dr. Szabo and his laboratory demonstrated the important roles of growth factors in the healing of other ulcers besides gastric ulcers, such as duodenal ulcers and large colonic lesions [49,51,52]. In transformed and stressed cells and in some tissues (e.g., brain), a single copy of *bFGF* encodes multiple gene products: 18 kDa and higher molecular weight (HMW) bFGF isoforms: ~21 and ~22 kDa in rodents; and ~22, ~23, and ~24 kDa in humans. The biologic roles of these HMW bFGF isoforms in vivo remain unknown. We demonstrated that in normal, uninjured rat gastric mucosa, bFGF is almost exclusively expressed as an 18 kDa isoform translated through a classical AUG (methionine) codon [53]. In contrast, in injured gastric mucosa of the rat, *bFGF* is preferentially translated to HMW *bFGF* isoforms through alternative CUG (leucine) initiation codon. Gastric mucosal injury caused in rats a significant increase in bFGF mRNA at 8 and 24 h vs. normal mucosa and a significant increase in bFGF protein after 24–72 h, mainly due to increased expression of ∼21 and ∼22 kDa HMW bFGF isoforms [53]. This demonstrated that gastric mucosal injury and repair triggers local activation of bFGF gene with preferential translation of HMW *bFGF* isoforms through a non-canonical CUG codon. That study uncovered CUG-initiated HMW *bFGF* translation as a novel regulatory mechanism operating in vivo during gastric injury healing [53].

## 8. Insulin-Like Growth Factor 1 (IGF-1) and GU Healing

**Figure 6:**
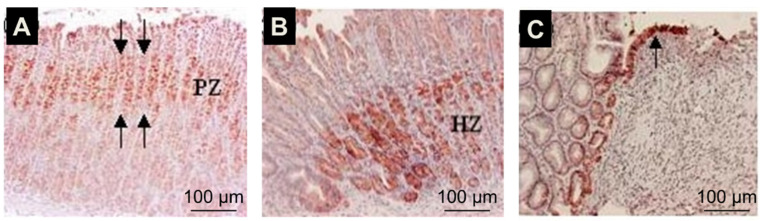
Gastric ulceration triggers local activation of IGF-1 in the ulcer margins. Immunostaining with an IGF-1-specific antibody and counterstaining with hematoxylin. In normal gastric mucosa, IGF-1 expression is localized to cells of the mucosal progenitor zone, PZ (**A**) (red staining). In ulcerated mucosa, IGF-1 expression is upregulated in the gastric ulcer margin healing zone, HZ, at day 6 (**B**,**C**). Epithelial cells migrating onto and re-epithelizing granulation tissue of gastric ulcers on day 6 also exhibited strong staining for IGF-1 (c, arrow). Control staining performed in the absence of primary antibody did not show any positive staining (reprinted with permission from [60]).

**Figure 7:**
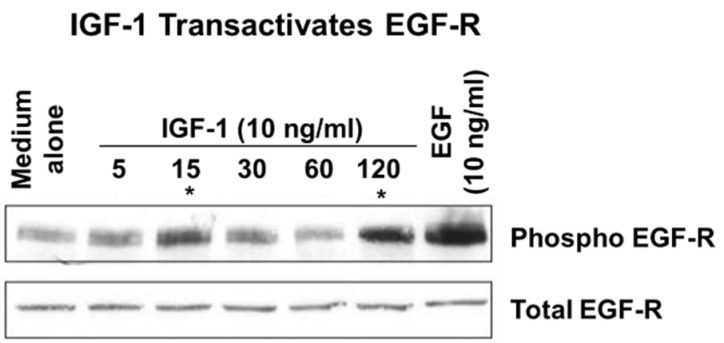
IGF-1 transactivates EGF-R in RGM1 cells. Treatment with IGF-1-phosphorylated EGF-R in a biphasic manner at 15 and 120-min. asterisk (*) denotes biphasic transactivation of EGF-R by treatment with IGF-1 at 15 and 120 min. (reprinted with permission from [14]).

IGF-1 also known as somatomedin C, is a protein structurally similar to insulin [54]. It plays an important role in childhood growth and has anabolic effects in adults. IGF-1 is produced by the liver as an endocrine hormone and in target tissues in a paracrine/autocrine fashion. Production is stimulated by growth hormone (GH). IGF-1 binds to IGF receptor-1 (IGFR-1), a tyrosine kinase membrane receptor. Activation of IGFR-1 by IGF-1 is important for epithelial and mesenchymal cell survival, growth, differentiation, and migration [55,56]. In the gastrointestinal tract, IGF-1 is secreted by salivary and other exocrine glands, and its receptor is present in epithelial cells of all segments of the rat gastrointestinal tract [57,58,59]. Our studies demonstrated that IGF-1 is upregulated in the gastric ulcer margin (Figure 6) and that exogenous IGF-1 promotes gastric re-epithelization, proliferation, and COX-2 expression via the PI3K pathway [60]. The upregulation of COX-2 and PI3K signaling may be a key mechanism in mediating IGF-1-induced cell proliferation and migration, both essential components of re-epithelialization and ulcer healing. It is unclear whether ulceration-induced increases in the expression of EGF and IGF-1 represent redundancy, a backup, or has a synergistic or an additive effect. Previous studies on the wounded keratinocyte monolayer demonstrated that EGF and IGF-1 influence keratinocyte shape differently. Although IGF-1 stimulated membrane protrusion and facilitated cell spreading, EGF also induced contraction of keratinocytes. The effects of each growth factor on keratinocyte shape were mediated by distinct signal transduction pathways: EGF stimulated the mitogen-activated protein kinase pathway, whereas IGF-1 stimulated the PI3K pathway. Our in vitro studies demonstrated that IGF-1 can transactivate EGF-R (Figure 7) [14]).

## 9. Temporal and Spatial Gene Expression during Ulcer Healing. Epithelial/Mesenchymal Interactions and Back-Ups

**Figure 8:**
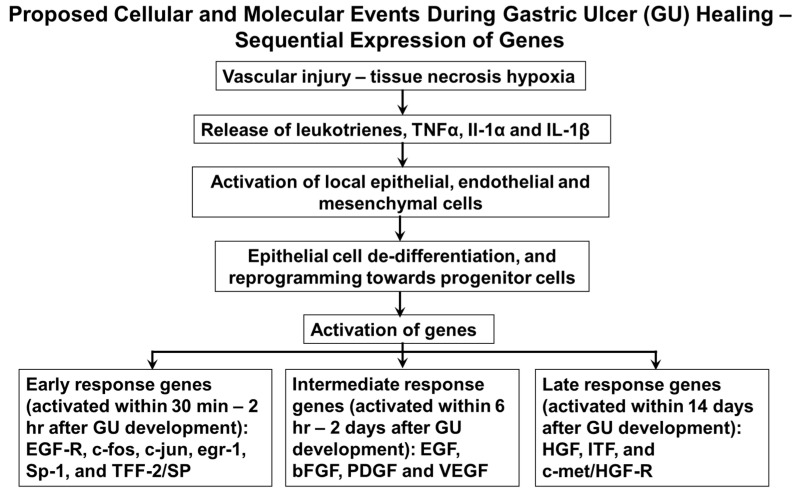
Proposed cellular and molecular events during gastric ulcer healing—sequential expression of genes.

The proposed molecular events during ulcer healing (sequential expression of genes) are summarized in Figure 8. As described above, gastric ulceration triggers the activation of a variety of growth factors and cytokines. Is it a redundancy, a backup, or an intended design of amplification or modulation? It should also be noted that in this complex healing environment, many growth factors are known to promote healing [41,46,60,61,62]. In this constellation of growth factors, it is difficult to determine the key signal for repair. Wong, Playford, and Wright have analyzed the sequential expression of various genes during gastric ulcer healing. Based on this analysis, they distinguished genes involved in the early response, EGF-R, c-fos, c-jun, egr-1, Sp-1, and TFF-2/SP, which are all activated shortly after ulcer formation (within 30 min–2 h); intermediate response genes, EGF, bFGF, PDGF, and VEGF (activated within 6 h–2 days); and late response genes, HGF, ITF, and c-met/HGF-R (activated within 14 days) [13]. It should be also pointed out that some of the growth factors are produced by epithelial cells, e.g., TFF, EGF, and TGFα; and others, e.g., PDGF, VEGF, HGF, and bFGF, by mesenchymal cells. Interestingly, in injury models, TFF upregulation can precede the upregulation of other growth factors, such as EGF and TGFα, suggesting that TFF peptides have the potential to initiate healing. Some of these growth factors affect not only mesenchymal but also epithelial cells, thereby providing coordination for local paracrine interactions between epithelial and mesenchymal components of ulcer healing and serving as a back-up system. Different mechanisms occurring at different times trigger chemical signals that modulate cell migration, cell proliferation, differentiation, and the synthesis and degradation of ECM proteins in orderly manner. These proteins, in turn, directly affect cellular events and modulate cell responsiveness to growth factors [1,5].

## 10. Cellular and Molecular Events in GU Granulation Tissue

**Figure 9:**
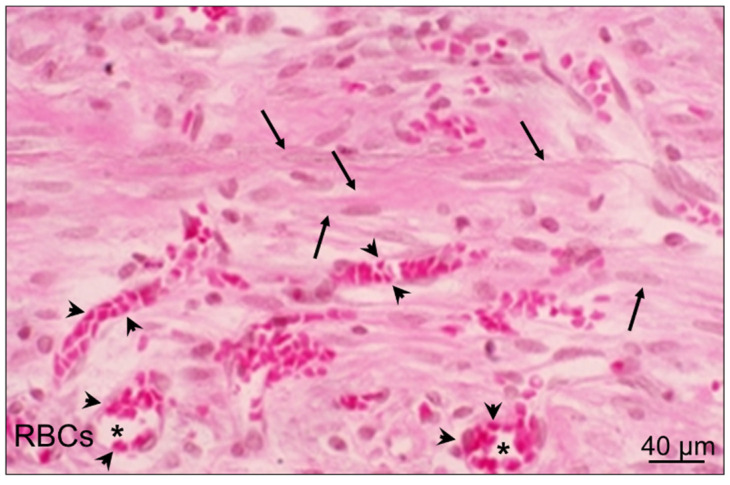
The granulation tissue at the ulcer base is the connective tissue component. This structure consists of fibroblasts, myofibroblasts (arrows), macrophages, and proliferating endothelial cells, forming blood vessels and microvessels (*) filled with erythrocytes (RBCs; arrowheads).

Granulation tissue develops at the ulcer base in 48–72 h. It is composed of fibroblasts, myofibroblasts, and vascular endothelial cells that proliferate, migrate, and form new blood vessels and microvessels through angiogenesis (Figure 9) [1]. The molecular events in granulation tissue that contribute to the mechanisms of gastric ulcer healing include the activation of genes encoding bFGF, FGF-R1,2,4, VEGF, and flk-1/KDR; Ang 1 and Ang 2; Tie 2 receptor; COX-2; SRF; NGF; SDF-1; and BMD-EPC via the activation of PI3K/Akt pathway.

The regeneration and restoration of blood vessels is a critical part of the healing of tissue injuries [1,5,6,7,11,63], including GUs, as addressed in our previous papers [64,65,66,67]. For a long time, revascularization in adult tissues was assumed to occur exclusively through angiogenesis—the sprouting of pre-existing endothelial cells (ECs) from areas bordering an injury. Alternatively, revascularization can be accomplished by the homing of bone marrow derived endothelial progenitor cells (BMD-EPCs) to injured tissues, resulting in the formation of new vessels via postnatal vasculogenesis [68,69,70,71,72,73,74,75]. It is unknown whether and to what extent BMD-EPC homing and postnatal vasculogenesis occur during GU healing and contribute to blood vessel regeneration in the ulcer scar.

Previous studies characterized the sequential events of angiogenesis in injured gastric mucosa and the causal relationship between VEGF, angiogenesis, and injury healing [1,7,67]. A recent study showed that in granulation tissue during GU healing, the angiogenesis originating from ECs of local blood vessels is complemented by the recruitment of BMD-EPCs into newly formed blood vessels, a hallmark of vasculogenesis [65]. These findings indicate that BMD-EPCs are incorporated into newly formed blood vessels of granulation tissue and thus contribute to and complement angiogenesis. The BMD-EPCs are attracted to granulation by activation and increased expression of stromal derived factor 1 (SDF-1) [76]. A potential advantage of vasculogenesis in combination with angiogenesis versus angiogenesis alone is the regeneration and formation of a more dense, more complete, and more elaborate microvasculature that provides better delivery of oxygen and nutrients to the regenerated mucosa and more efficient removal of toxic metabolites [64,65].

## 11. VEGF and Angiogenesis

VEGF, initially identified as a vascular permeability factor secreted by some tumors, was found in 1989 by Ferrara and Henzel to be identical to a factor in pituitary follicular cells. They purified, cloned, and named this factor VEGF. Ferrara et al. also identified, in 1992, VEGF receptor, as Fms-like tyrosine kinase-1 (flt-1) [77]. VEGF is a fundamental regulator of angiogenesis and is an endothelial cell-specific mitogen because its receptors are primarily restricted to endothelial cells [61,77,78]. VEGF binds to at least two specific receptors, VEGF-R1 and VEGF-R2, which are expressed mainly on endothelial cells, and initiates the phosphorylation of numerous cytosolic proteins involved in signal transduction that trigger endothelial cell proliferation, migration, and microvascular tube formation and angiogenesis [61,77,78]. VEGF production is stimulated by PDGF, TGFβ1, bFGF, cytokines, nitric oxide, and E-series prostaglandins [61,77,78]. Hypoxia is one of the best characterized stimuli for the induction of VEGF production by a variety of cells and tissues. Hypoxia triggers the accumulation of a hypoxia inducible factor (HIF-1) that binds to and activates the VEGF gene promoter [61,77]. The essential role of angiogenesis in ulcer healing is underscored by the fact that the stimulation of angiogenesis in granulation tissue by treatment with exogenous bFGF, PDGF, or VEGF dramatically accelerates the healing of experimental gastric and duodenal ulcers in rats [49,51,79]. Additionally, a local therapy with VEGF plus angiopoietin 1 cDNAs or with serum response factor (SRF) plasmid dramatically accelerates gastric ulcer healing and improves the quality of mucosal restoration within ulcer scars [79,80]. Importantly, a single local submucosal injection of naked DNA encoding VEGF165 and Ang1 into a GU site significantly increased neovascularization and accelerated GU healing. A neutralizing anti-VEGF antibody significantly reduced the acceleration of ulcer healing resulting from the treatment. Co-injection of two plasmids encoding rhVEGF165 and rhAng1 resulted in the formation of more mature vessels and more complete restoration of gastric glandular structures within the ulcer scar. This demonstrated an essential role for VEGF and enhanced angiogenesis in GU healing.

## 12. Novel Roles of SRF in Re-Epithelialization and VEGF Induced Angiogenesis

SRF, a transcription factor, also plays important roles in gastric ulcer healing [80]. SRF was discovered in 1986 by Treisman, who demonstrated that serum induces in fibroblasts the expression of *c-fos* through activation of a transcription factor, SRF, which binds to the serum response element (SRE) in the *c-fos* promoter [81]. SRF has been shown in several types of cells (e.g., fibroblasts and muscle cells) to regulate the expression of numerous genes, the products which are important for a variety of cellular activities (reviewed in [82]). SRF is activated by serum, growth factors, cytokines, and other mitogenic stimuli [82]. In turn, SRF activates immediate early genes such as *c-fos*, egr-1/2, *cyr61*, and cytoskeletal genes. By activating these genes in response to external stimuli, such as growth factors, tissue injury, and cellular stress, SRF triggers proliferation of smooth muscle cells and fibroblasts, and differentiation of fibroblasts into myofibroblasts [80,81,82,83,84].

It has been demonstrated that serum itself induces a number of genes that are usually activated during tissue injury and wound healing, including pro-inflammatory regulators (e.g., interleukin-1α, interleukin-6, and COX-2), immediate early genes (e.g., *c-fos*, *egr-1*, and *jun B*), and angiogenic factor genes (e.g., bFGF and vascular endothelial growth factor-VEGF) [85]. This is not surprising because in vivo epithelial cells are directly exposed to serum only after vascular injury and wounding. Therefore, the cells are programmed to interpret the serum exposure not only as a general mitogenic stimulus, but also as a specific signal signifying a wound [80,85]. Hence, the features of the transcriptional program of a cell in response to serum exposure in vitro may resemble many aspects of the wound healing process in vivo.

## 13. The Role of SRF in GU Healing

As growth factors, cytokines, and serum extravasated during tissue injury and ulceration can activate SRF, we examined whether gastric ulceration induces SRF expression [80] and obtained gastric specimens sequentially after GU induction in rats for SRF mRNA and protein expression, and for immunohistochemistry. We also examined the role of SRF in gastric ulcer healing by locally injecting an SRF expression plasmid into the GU base [80]. To determine the cellular mechanisms of SRF action, we examined the effect of SRF overexpression on actin dynamics, cell migration, and proliferation in rat gastric epithelial cells (RGM-1) and smooth muscle cells (A7R5). To determine the clinical relevance, we examined SRF expression in human GU specimens [80]. These studies showed that gastric ulceration activates the expression of SRF in the epithelial cells of the ulcer margin and regenerating glands, and in myofibroblasts and smooth muscle cells of granulation tissue. In human GUs, SRF was upregulated similarly to in rat GUs. This demonstrated direct human relevance of SRF [80]. In vivo therapy with SRF significantly accelerated GU healing in rats and promoted re-epithelialization and muscle restoration [80]. Overexpression of SRF in RGM1 and A7R5 cells accelerated these cells’ migration and proliferation, promoted actin polymerization and the formation of stress fibers, and activated the expression of immediate early genes [80]. Since epithelial cell migration during GU healing is stimulated by EGF, we examined the effect of EGF on the migration of SRF-transfected cells compared with control cells. In the SRF transfected RGM-1 cells at baseline (without EGF treatment), there was a 7-fold increase in migration in comparison to cells transfected with the plasmid without the SRF insert (control). Treatment with exogenous EGF induced an additional 17-fold increase in migration of the SRF-transfected RGM-1 cells [80]. This study also showed that SRF was crucial for the polymerization of G-actin into F-actin and the formation of actin stress fibers [80]. In a separate study we demonstrated that SRF is a downstream mediator of VEGF signaling in endothelial cells and a critical, sine qua non requirement for VEGF-induced angiogenesis [86]. Knockdown of SRF protein levels in human and rat endothelial cells abolished VEGF-induced in vitro angiogenesis, impaired endothelial cell migration and proliferation, and inhibited VEGF-induced actin polymerization and immediate early gene expression [86]. Injection of SRF antisense expression plasmid into GUs in rats significantly inhibited in vivo angiogenesis in granulation tissue [80]. Mechanistically, this study also revealed that VEGF promotes SRF expression and nuclear translocation and increases SRF’s binding to DNA in endothelial cells through both Rho-actin and MEK-ERK dependent signaling pathways. These studies showed that SRF is a critical switch regulator of GU healing by promoting re-epithelialization, angiogenesis, and muscular structure restoration during GU healing [80].

## 14. A Novel Role of Nerve Growth Factor (NGF) in GU Healing

**Figure 10:**
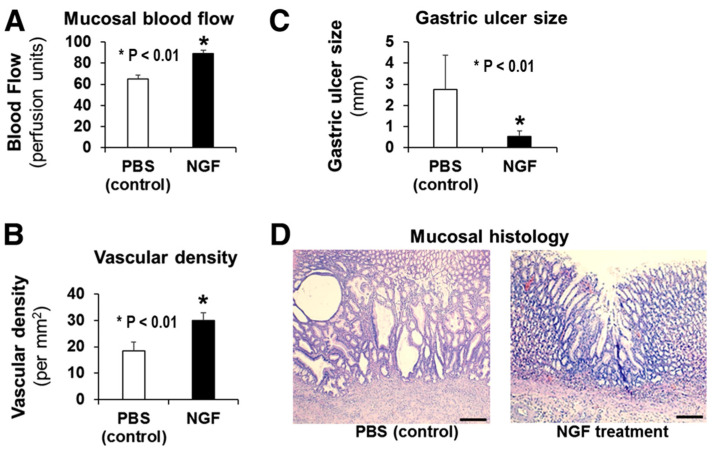
Local NGF therapy of GUs in aging rats increases angiogenesis, accelerates gastric ulcer healing, and improves the quality of mucosal regeneration. In aging rats, either PBS (control) or NGF (100 μg/kg body weight in 200 μL PBS) was locally injected to the ulcer base after GU induction. Mucosal blood flow, microvessel density, GU size, and mucosal regeneration were examined 3 weeks after GU induction. Local NGF treatment of GUs in aging rats (**A**) significantly increased mucosal blood flow in GU scars/margins by 37% compared to the control, and (**B**) increased vascular density in granulation tissue at the base of the GU scar/margin by 62% compared to the control, both showing increased angiogenesis. (**C**) NGF therapy accelerated GU healing, as reflected by the >5-fold reduction in GU size. (**D**) Mucosal histology showed better regeneration of gastric mucosa in GU scars of NGF-treated rats compared to the poor, disorganized, and abnormal regeneration (e.g., large, dilated, irregular abnormal glands) in the control group (reprinted with permission from [96]).

NGF was discovered in the early 1950s by Rita Levi-Montalcini as a factor critical for the growth and survival of neurons [87,88,89]. Subsequent work by her group and research by Shooter and his team made landmark contributions to the field of NGF [90,91]. The role of NGF in gastric ulcer healing had not been explored before. Our studies on the quality of ulcer healing demonstrated, in the scars of healed GUs in rats, a virtual absence of CRGP-positive nerve fibers, both in the submucosal scar and in the mucosa [28]. This suggested an absence or reduced expression of NGF. In recent years, NGF has gained attention for its actions that extend beyond the promotion of neuronal survival and outgrowth [92,93]. One such “non-canonical” action of NGF is the ability to promote angiogenesis in brain capillary ECs [62]. Our recent study demonstrated that NGF is expressed in gastric ECs and stimulates angiogenesis independently of VEGF [94]. That study also showed that silencing of NGF in gastric ECs abolishes angiogenesis, and this effect can only be reversed by exogenous NGF, but not VEGF [94]. In vivo studies in rat GUs demonstrated that local NGF therapy in young rats increases angiogenesis, accelerates GU healing, and improves the quality of mucosal regeneration [95]. Our studies also showed that NGF expression is reduced in vivo in gastric endothelial cells of aging rats, and local NGF therapy of GUs in aging rats increases angiogenesis, accelerates ulcer healing, and improves mucosal regeneration in ulcer scars (Figure 10) [96].

## 15. Summary and Future Directions

As described above, several growth factors accelerate and improve GU healing. Even a single local injection of some growth factors, e.g., EGF, TGFα, HGF, and NGF, or a single local submucosal injection into GU area of VEGF165 and Ang1 cDNAs significantly accelerated healing of experimental GU. Conversely, neutralizing antibodies significantly reduced the acceleration of ulcer healing resulting from these treatments, clearly demonstrating a cause-and-effect relationship.

Importantly, local administration of NGF protein to the GU sites in rats accelerated ulcer healing, increased angiogenesis, and improved the quality of mucosal regeneration. Specifically, mucosal histology showed better regeneration of gastric mucosa in the GU scars of NGF-treated rats compared to the poor, disorganized, and abnormal regeneration (forming large, dilated, irregular abnormal glands) in the control group (Figure 10) [96]. Additionally, a single local injection of VEGF165 plus Ang1 cDNAs with a limited duration of target gene expression significantly increased neovascularization, accelerated ulcer healing, and resulted in the formation of more mature vessels and more complete restoration of gastric glandular structures within the ulcer scar [79]. Inhibition of accelerated healing with a neutralizing anti-VEGF antibody indicated an essential role for VEGF and enhanced angiogenesis in ulcer healing. These findings point to a very important topic: the potential of GU treatments using growth factors and/or their combinations for improving the quality of ulcer healing. In the past, evaluations of GU healing were based on visual endoscopic examinations in patients or the measurements of ulcer size in experimental studies. Such an approach resulted in the assumption that the mucosa of macroscopically “healed” GU is fully regenerated. However, detailed studies demonstrated that the mucosa of macroscopically “healed” experimental GUs has prominent histologic and ultrastructural abnormalities, including reduced height, abnormal distorted gastric glands, poor differentiation, and disorganized vasculature [9,97]. These abnormalities might interfere with mucosal defense and may be the basis for ulcer recurrence in the scars of healed ulcers [9,97]. More recent studies confirmed this contention by showing prominent histologic, structural, and gene expression abnormalities in macroscopically healed gastric epithelium [98,99]. These studies also showed that the regenerated epithelium after ulcer healing remains abnormal for months after healing and suggested that the sites of ulcer scars are where ulcer recur.

Thus, the quality of GU healing reflected by the completeness of regeneration of mucosal structures is particularly important for the prevention of GU recurrence. Since a local treatment of GU site with some growth factors, as described above, improves the quality of healing, their use can provide a clear advantage to GU, and perhaps other GI ulcer treatments.

## 16. Potential Future Directions—Therapy of GU with Local Administration of Growth Factors?

The treatment GU using endoscopic needle injections of individual growth factors or their combinations into ulcers could potentially be used for chronic or non-healing human GUs. Although therapeutic use of growth factors has great promise in regenerative medicine, translation of growth factors into clinical treatments has limitations that include low protein stability and low yields of recombinant expression. These limitations could be overcome by using combinatorial protein engineering to improve growth factor stability, and increase GF expression, biodistribution, and tissue half-lives; or by altering their cell or receptor binding affinity. Additionally, the use tissue engineering with biomaterial-based systems mimics the natural extracellular matrix for effective spatial and temporal delivery of growth factors to cell surface receptors [100]. To this end, temporally controlled growth factor delivery from a self-assembling peptide hydrogel and electrospun nanofiber composite scaffold has been developed [100]. It provides a way to stabilize and control the delivery of growth factors necessary for optimal tissue repair and healing. Another promising group of treatments for accelerating the healing of GI ulcers are the recently developed small molecule FAK activators that promote human intestinal epithelial cell migration, monolayer wound closure, and mouse ulcer healing [101,102,103]. Regarding potential clinical translation, several growth factors, e.g., PDGF and recombinant human EGF, are used to treat diabetic foot ulcers by local injection into the wound sites, and several other growth factors are in clinical trials for wound healing [100,104,105,106,107,108].

## Data Availability

Not applicable.

## Abbreviations

AKT	serine-threonine protein kinase
Ang	angiopoietin
AR	Amphiregulin
bFGF	basic fibroblast growth factor
BMD-EPCs	bone marrow derived endothelial progenitor cells
BTC	Betacellulin (BTC)
COX-2	cyclooxygenase 2
ECs	endothelial cells
EGF	epidermal growth factor
EGF-R	epidermal growth factor receptor
EPR	Epiregulin
ERK	extracellular regulated kinase
FGF	fibroblast growth factor
GT	granulation tissue
GU	gastric ulcer
HB-EGF	heparin-binding EGF-like growth factor
HGF	hepatocyte growth factor
HIF	hypoxia inducible factor
Hp	Helicobacter pylori
IGF-1	Insulin-like growth factor 1
IL-1α	interleukin-1 alpha
IL-lβ	interleukin-1 beta
KGF	Keratinocyte growth factor
MAPK	mitogen activated protein kinase
NGF	nerve growth factor
NRG1-4	neuregulins 1-4
NSAIDS	non-steroidal anti-inflammatory drugs
PDGF	platelet derived growth factor
PGs	prostaglandins
PI-3K	Phosphatidylinositol 3-kinase
PLCγ	Phospholipase C gamma
Rho/Rac	GTPases
RT-PCR	reverse transcription polymerase chain reaction
SDF-1	stromal derived factor 1
SRE	serum response element
SRF	serum response factor
TGFα	transforming growth factor alpha
TGFβ	transforming growth factor beta
TFF	Trefoil factor
TNFα	tumor necrosis factor alpha
UACL	ulcer-associated cell lineage
VEGF	Vascular endothelial growth factor
